# First passage time distribution in heterogeneity controlled kinetics: going beyond the mean first passage time

**DOI:** 10.1038/srep20349

**Published:** 2016-02-08

**Authors:** Aljaž Godec, Ralf Metzler

**Affiliations:** 1 Institute of Physics & Astronomy, University of Potsdam, 14776 Potsdam-Golm, Germany; 2 National Institute of Chemistry, 1000 Ljubljana, Slovenia; 3 Department of Physics, Tampere University of Technology, FI-33101 Tampere, Finland

## Abstract

The first passage is a generic concept for quantifying when a random quantity such as the position of a diffusing molecule or the value of a stock crosses a preset threshold (target) for the first time. The last decade saw an enlightening series of new results focusing mostly on the so-called mean and global first passage time (MFPT and GFPT, respectively) of such processes. Here we push the understanding of first passage processes one step further. For a simple heterogeneous system we derive rigorously the complete distribution of first passage times (FPTs). Our results demonstrate that the typical FPT significantly differs from the MFPT, which corresponds to the long time behaviour of the FPT distribution. Conversely, the short time behaviour is shown to correspond to trajectories connecting directly from the initial value to the target. Remarkably, we reveal a previously overlooked third characteristic time scale of the first passage dynamics mirroring brief excursion away from the target.

How fast does the amplitude or position of a random process reach a given threshold value (target) for the first time? This so-called first-passage time (FPT)^1^,^2^ is central to the description of the kinetics in a large variety of systems across many disciplines, including diffusion controlled chemical reactions^3^, signalling cascades in biological cells^4^,^5^, transport in disordered media^6^ including the breakthrough dynamics in hydrological aquifers^7^, the location of food by foraging bacteria and animals^8^,^9^ up to the global spreading of diseases^10^,^11^ or stock market dynamics^12^. In the following we discuss the FPT problem in the language of the diffusion of a physical particle in position space.

Contrasting their diverse phenomenology, the kinetics in stochastic systems such as the above can often be rephrased in terms of the simplest—but extensively studied—random walk. In unbounded space the FPT statistics of the random walk—or in fact its diffusion limit—are heavy-tailed, giving rise to a diverging mean FPT (MFPT)^1^. Heavy tails are in fact common when it comes to persistence properties of infinite systems^13^. Conversely, a finite system size suppresses the heavy tails, effecting an exponential long time statistic and thus a finite MFPT, which becomes a function of the system size and dimensionality^1^,^14^.

Generically we distinguish two universality classes of the statistic of the global FPT (GFPT)—the FPT averaged over all initial positions inside the domain of interest—for a variety of dynamics in translation invariant media, depending on the nature of how the the surrounding space is explored^14^,^15^,^16^: in the case of non-compact exploration leaving larger regions of the domain unexplored such as in diffusion in three spatial dimensions, the initial separation between the walker and its target does not play a dominant role^15^. The situation is reversed in the case of compact exploration of space such as for the diffusion on fractal geometries. Now the initial separation dominates and leads to so-called geometry-controlled kinetics^15^. Note that for the statistic of the GFPT the non-trivial dependence of the FPT statistics on the initial position is effectively integrated out.

Many studies of FPT kinetics concentrate on the determination of the MFPT or the GFPT which often are useful to determine the rough time scale of the underlying process. However, even for Brownian motion the distribution of FPTs shows a very rich phenomenology and—depending on the location of the target—may exhibit highly non-uniform FPT kinetics^17^,^18^,^19^. Under certain conditions, any two independent first passage trajectories are most likely to be significantly different. In such cases the MFPT—albeit finite—is not a precise parameter to describe the FPT statistic^17^,^18^,^19^,^20^. Quite generally, the first passage statistic of Brownian motion has the generic asymptotic behaviour^18^


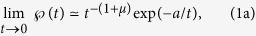



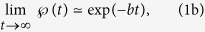


where *μ* is the so-called persistence exponent^13^ and *a* and *b* are dimension and geometry specific parameters. Eq. (1a) encodes the fact that it takes a finite minimum time to reach the target followed by a power law decay of FPTs on a time scale on which the searcher does not yet feel the presence of the boundary. In addition, Eq. (1b) states that the searcher will eventually find the target in a finite system of linear dimension *R* on a time scale up to 
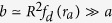
, where 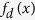 is a dimension dependent function, which diverges as the target radius 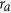 goes to zero in two and three dimensions^18^.

The above results hold for translation invariant systems. Yet, numerous real systems such as biological cells are spatially heterogeneous and therefore display fundamentally different dynamics^21^,^22^,^23^,^24^. Various aspects of diffusion in heterogeneous media have already been addressed^25^,^26^,^27^,^28^,^29^,^30^,^31^ but the rôle of spatial heterogeneity in the FPT statistics beyond the MFPT^32^ remains elusive. Moreover, the results in ref. 32 suggest that the MFPT in (hyper)spherically symmetric domains is apparently independent of indirect trajectories, these are those that interact with the confining boundary, in contrast to direct trajectories, that head swiftly to the target.

Here we present exact results for the full FPT statistics in a simple heterogeneous model system. Based on a rigorous asymptotic analysis of the FPT statistics of Brownian motion in a confined spherically symmetric domain with a piece wise constant diffusion coefficient, see Fig. 1 and ref. 32, we here demonstrate the emergence of a new time scale in the FPT dynamics, which is controlled by the spatial heterogeneity. More precisely, we prove that the intermediate time power law asymptotics in Eq. (1a) breaks down in a sufficiently heterogeneous medium. For such *heterogeneity-controlled kinetics* we derive analytical asymptotic results for the short, intermediate, and long time FPT statistics for an arbitrary degree of heterogeneity. We also quantify the most likely (typical) FPT and the width of the FPT distribution. We demonstrate that the MFPT is dominated by long and unlikely indirect trajectories, while the overall relative contribution to the MFPT of the latter remains coupled to the most likely, direct trajectories. Finally, we discuss the implications of our results for more general systems.

## Results

### System setup and general result

We consider a spherically symmetric and potential free system with a perfectly absorbing central target of finite radius 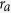 and a perfectly reflecting boundary at radius 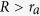
32. The system is in contact with a heat bath at constant and uniform temperature *T*. The particle experiences a space dependent friction 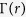 originating from spatial variations in the long range hydrodynamic coupling to the motion of the medium^33^. We focus on the high friction limit corresponding to overdamped motion and assume that the particle diffuses with the isotropic position dependent diffusion coefficient 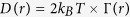. Simultaneously, the diffusing particle experiences the fluctuation induced thermal drift 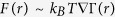 ensuring thermodynamic consistency in the sense that 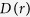 has a purely stochastic origin and does not reflect any heterogeneity in the entropic potential of mean force^34^. More precisely, in the absence of the target the system relaxes to the correct Boltzmann-Gibbs equilibrium—a spatially uniform probability. The thermodynamically consistent theory of diffusion in inhomogeneous media^34^ corresponds to the so-called kinetic interpretation of the underlying multiplicative-noise Langevin equation; see, for instance, refs 35,36.

We are interested in the evolution of the probability density function 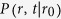 in dependence on the particle radius 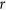 at time 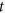 after starting from the initial radius 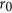 at 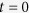. Due to the symmetry of the system the angular co-ordinate is not of interest, and we average over the space angle. The diffusion equation governing the radial probability density function 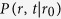 is then given by


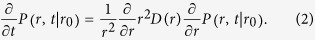


**Figure 1:**
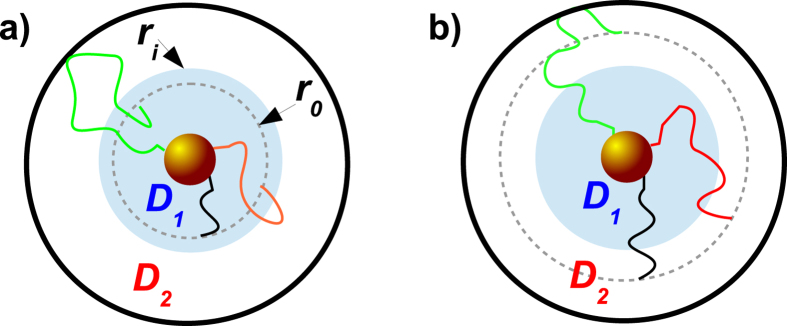
Schematic of the model system with absorbing, finite central target of radius *r*_*a*_. The concentric shell of radius 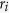 separates regions of different diffusivities 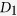 and 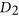. The initial radius of the particle (dashed line) is 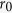, and the outer shell at radius *R* is reflective. The black and red lines denote direct trajectories and the green line denotes an indirect trajectory, see text for details.

In our analysis we consider the particular case of a piece wise constant diffusion coefficient of magnitude 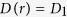 for 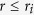 and 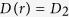 otherwise—see also Fig. 1. Physically, this form should be viewed as an ideal limit of a two phase system with a sharp interface. A similar limit of an infinitely sharp interface can be taken for the Langevin equation of the process, as well.

The exact solution for the Laplace transform 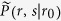 was derived in ref. 32. From this result the Laplace transform of the FPT density is obtained from the corresponding probability flux into the target, 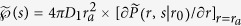. We introduce dimensionless variables 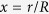 for the particle position, 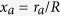 for the target radius, 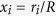 for the interface radius, and 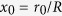 for the initial particle position, as well as express time in units of 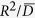, where 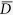 is the spatial average of 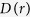. The exact result then reads


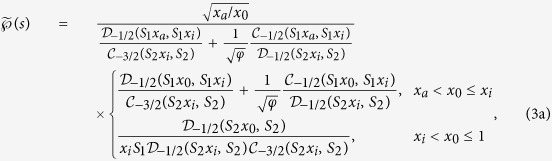


where we introduced the ratio 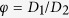 of the diffusivities along with the abbreviation 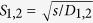 and the auxiliary functions


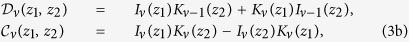


where 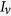 and 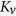 denote the modified Bessel functions of the first and second kind, respectively^37^.

In order to allow for a meaningful comparison of the FPT kinetics at various degrees 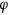 of heterogeneity we introduce a constraint on the conservation of the spatially averaged diffusion coefficient, 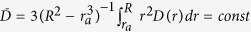, for a detailed discussion of this choice see ref. 32. In addition, in the general case the absolute value of 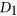 only sets the time scale of the problem, whereas 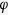 gives rise to the qualitative changes in the FPT kinetics in our heterogeneous system. With the introduced average diffusivity constraint both diffusivities are fully determined by 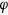 and 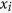, that is, 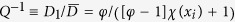. Note, however, that the constraint does not introduce any additional information which would affect the qualitative picture of our results. The general case for arbitrary 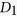 and 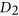 is recovered trivially by treating *Q* as an independent parameter or by replacing 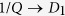 and 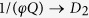.

Eq. (3a) is the starting point of our asymptotic analysis. In addition, in order to validate the analytical results we numerically invert 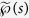 using the fixed Talbot method^38^.

### Short time asymptotic

Starting from the central result (3a) the short time behaviour of the FPT distribution 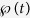 is obtained from the asymptotic behaviour of the respective modified Bessel functions for large argument (large Laplace variable)^37^. First it can be shown for 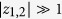 that


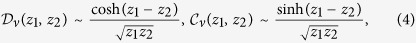


which is valid for 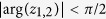 and 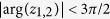, respectively. Combined with Eq. (3a) we obtain a limiting expression for 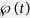 which can be inverted exactly—by performing the contour integral within the domain of validity of Eq. (4)—leading us to the Lévy-Smirnov density


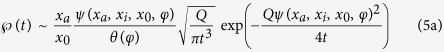


where we introduced


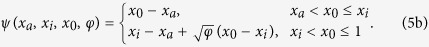


We take 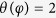 if 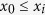 and 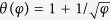 otherwise. It is easy to see that all 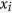-dependent terms vanish for a homogeneous systems with 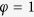. Note that Eq. (5a) obeys the generic behaviour given in Eq. (1a) with a persistence exponent 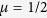 and 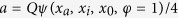. Moreover, Eqs (5a) and (5b) have an intuitive physical meaning: they show that the earliest FPTs will be observed on a time scale on which the particle diffuses over a distance corresponding to the initial separation to the target, with the respective diffusion coefficients. Moreover, we note that the FPT density to a *finite* radius 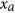 for three dimensional radial Brownian motion in our heterogeneous system obeys the 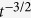 scaling Sparre Andersen theorem which needs to generally hold for one dimensional Markov processes with symmetric jumps^39^,^40^. Most importantly, Eq. (5a) is *independent* of 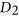 for 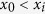. In other words, the first passage behaviour of particles released inside the interface radius 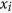 is dominated by trajectories, which head straight for the target and do not venture into the outer part of the system. These are the *direct trajectories* introduced in ref. 32, see also below.

**Figure 2:**
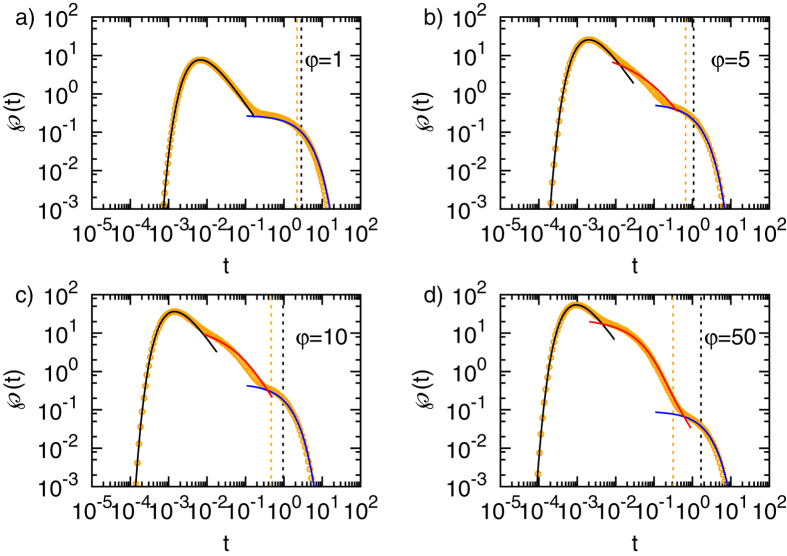
FPT densities for various degrees of heterogeneity *φ* and target radius *x*_*a*_ = 0.1, interface radius *x*_*i*_ = 0.5, and initial radius *x*_0_ = 0.3. In this Figure the particle starts inside the inner region. The symbols denote the results of the numerical inversion of Eq. (3a). The black lines correspond to the short time limit (5a), the blue lines denote the long time asymptotics given by Eq. (7). The red lines correspond to the intermediate time asymptotics in Eq. (11a). The dashed vertical lines denote the corresponding MFPT from 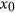 (orange) and from the outer boundary at 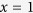 (black), respectively.

**Figure 3:**
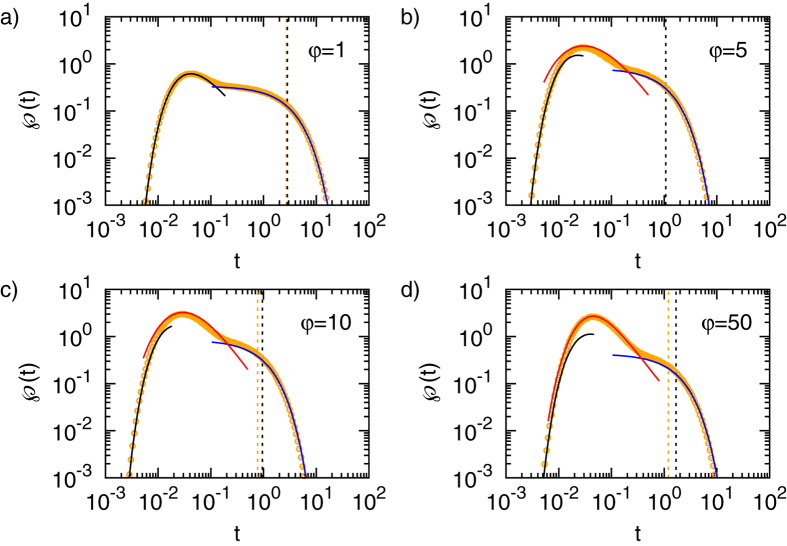
FPT densities for various degrees *φ* of heterogeneity and parameters *x*_*a*_ = 0.1, *x*_*i*_ = 0.4 and *x*_0_ = 0.6. In this Figure the particle starts in the outer region of the system. The symbols denote the results from numerical inversion of Eq. (3a). The black lines correspond to the short time limit (5a) and the blue line denotes the long time asymptotics (7). The red line represents the intermediate time asymptotics (11b).

The FPT densities for various degrees of heterogeneity 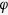 are shown in Figs 2 and 3, corresponding to initial positions 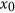 in the inner and outer regions, respectively. Note the excellent agreement between the exact numerical result for 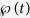 and the short-time asymptotics in Eq. (5a) as seen from comparison of the symbols with the black lines.

### Long time asymptotic

The long time asymptotic behaviour of the FPT distribution 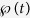 is obtained from Eq. (3a) by using the expansions of the respective modified Bessel functions for small argument (small Laplace variable *s*)^37^. Here we strictly note that corrections to leading order terms in 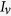 and 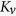 must be retained in order to obtain the correct behaviour of 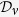 and 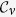 for small *s*. We find that


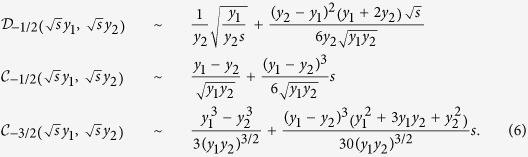


Combining Eq. (6) with Eq. (3a) we invert the Laplace transform exactly, yielding the exponential density


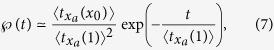


where the symbol 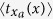 denotes the MFPT to 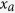 if starting from *x*. Its result is given by


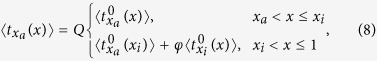


where 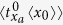 denotes the MFPT from 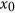 to 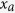 in a *homogeneous* sphere with unit radius and with unit diffusion coefficient,


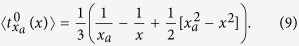


As before, all 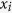-dependent terms vanish for a homogeneous system.

Eq. (7) has the generic form of the exponential long time tail (1b) of the FPT distribution in a finite system. From Eq. (7) we identify the inverse of the characteristic time as 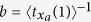. Moreover, Eq. (7) has an intuitive meaning: it demonstrates that first FPTs are exponentially unlikely beyond a time scale corresponding to the MFPT to arrive at the target from the external boundary. In turn, the long time exponential region evidently corresponds to trajectories, which are reflected from the external boundary. Therefore, for 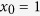 we can no longer distinguish between direct and indirect trajectories. Note the excellent agreement between the exact numerical result for 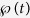 and the long time asymptotics (7) in Figs 2 and 3 as shown by the blue lines.

Moreover, we emphasise another observation. For a homogeneous system with 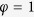 the short and long time asymptotics together fully describe the FPT density. Put differently the overlap region between the regimes is extremely narrow. This holds true in general up to some critical heterogeneity 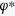, which will be specified in the following section. Beyond this value 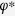 a new time scale emerges, as seen in Figs 2b–d and 3b–d) which is not captured by the the short and long time asymptotics and does *not* correspond to an overlap regime, as we now explain.

### Emergence of a new time scale

Here we focus on the regime 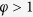 when the inner region has the higher diffusivity. This is the scenario which we would naively expect to enhance the FPT kinetics. The opposite case 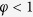 is physically less interesting but can be obtained analogously to the steps presented below. Note that in this regime a resolved third time scale does not exist because short excursions into the outer region are always faster. However, as 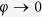 the correct short time asymptotics is obtained in the limit 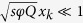 and 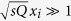, and these are in general not of the Lévy-Smirnov type (5a). Considering the two different types of argument in Eq. (3a), 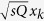 and 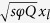 it becomes obvious that an additional separation of time scales occurs in the limit 
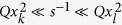
, where *k*, *l* stand for the different indices used in our model and depend on the starting position relative to the interface position. In other words, there exists a time scale separation between direct trajectories corresponding to the short time asymptotic (5a) and reflected trajectories accounted for by the long time asymptotic (7).

This new time scale corresponds to trajectories, which are much longer than the direct ones yet much shorter than the reflected ones. In such trajectories the particle ventures into the outward direction away from the target with respect to its initial position. However, this excursion is much shorter than the average time needed to reach the interface. The result are terms of mixed order in *s* having the form


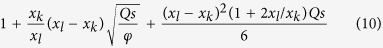


with 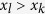. In this limit the second and third terms are comparable and both need to be explicitly considered in the analysis. The exact intermediate time asymptotic forms of 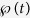 can be derived rigorously and read


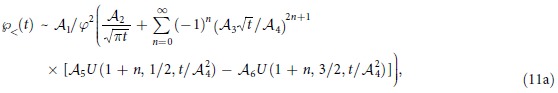


when 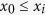 and


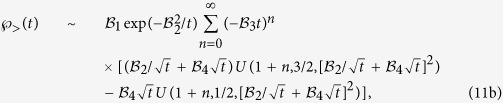


when 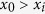 as well as for 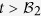. Here, 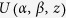 denotes Tricomi’s confluent hypergeometric function^37^. The coefficients 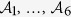 and 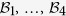 are given in Eqs (20) and (21) in the Appendix. Details of the calculation will be reserved for a separate longer publication. Both forms, Eq. (11a) and Eq. (11b) hold for 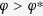 with


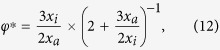


independently of 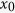. The transition as 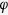 crosses the critical value 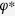 is discontinuous, the factor involved changing from 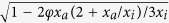 to 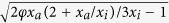, and the functional dependence on *t* changes concurrently as well. Below 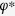 the FPT distribution 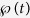 is completely specified in terms of Eq. (5a) and Eq. (7). This is what we may call a *threshold heterogeneity*. Moreover, while Eq. (11a) does not reduce to a simple form as 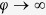, Eq. (11b) reduces to a Lévy-Smirnov density,


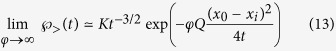


with a computable prefactor *K* different from the quantity 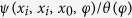 in Eq. (5a). Other than that, Eq. (11b) continuously interpolates between the two different Lévy-Smirnov densities Eqs (5a) and (13) as 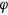 increases. Intuitively, this latter limit demonstrates the fact, that reaching the interface from 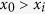 becomes rate limiting for large 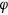. We also note that the infinite series in Eqs (5a) and (13) converge fast, and in numerical evaluations it suffices to consider the first 10–15 terms for any value of 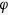. The intermediate asymptotic formulas are compared to the exact numerical result in Figs 2 and 3, as shown by the symbols and the red line. We find a good agreement, which intuitively depends on the separation of time scales and hence improves for large 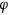. Strikingly, for 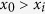 the intermediate time regime includes the *most likely* FPTs. Therefore, most likely trajectories are indeed direct, as we anticipated already in 32.

**Figure 4:**
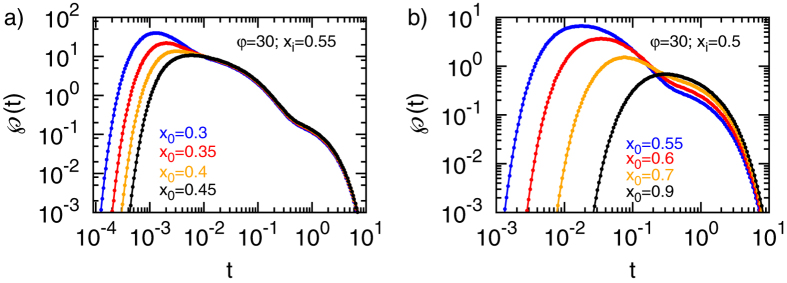
FPT densities for target radius *x*_*a*_ = 0.1 and various combinations of the starting position *x*_0_ and the interface radius *x*_*i*_, as denoted in the panels.

To gain more intuition on how exactly the time scale separation arises we plot the exact numerical result for the FPT distribution 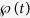 at 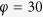 for different initial positions, as depicted in Fig. 4. Starting in the inner region the scale separation emerges as we continuously move the initial position towards the target. Conversely, if starting in the outer region a scale separation emerges as we continuously move the initial position away from the reflecting surface. The necessary and sufficient requirement for heterogeneity controlled kinetics is therefore a large heterogeneity and a corresponding existence of two length scales inwards and outwards from the initial position towards the closest boundary or interface.

### Mean first passage times are not typical

We now quantify the most likely or typical FPT times, i.e., those which occur most frequently. Already from Figs 2 and 3 it is apparent that there is a large discrepancy in the likelihood of typical and mean FPTs, compare the maximum of 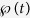 with the dashed vertical line denoting the MFPT. In many cases the most likely FPT is the more relevant quantity. For instance, consider a certain species of bacteria, in which genetic regulation can be viewed as an FPT problem^5^. When we compare the fitness of an individual bacteria in a colony, those who are among the first to respond to an external challenge will be of advantage. Similarly, those predators that first discover a prey have a larger chance of survival.

The MFPT was defined in the previous section, while the typical FPT is corresponds to the extremum 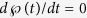. In the case 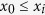 we find that


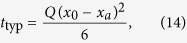


whereas in the case 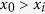 we obtain closed form expressions only in the limits 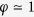 and 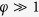, these being


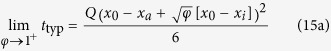



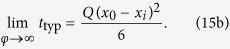


For general values of 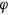 the typical FPTs are computed numerically.

**Figure 5:**
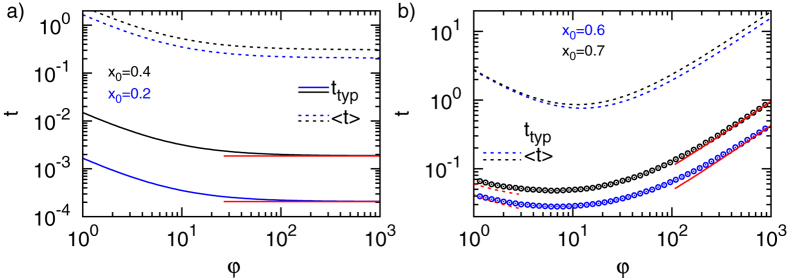
Comparison of the MFPT and the most likely FPT for two different initial conditions and target radius *x*_*a*_ = 0.1: (**a**) 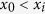 for 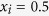 and (**b**) 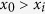 for 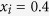. The full and dashed black and blue lines denote the MFPT and the most likely FPT, respectively. The red lines correspond to Eq. (15b).

The typical and mean FPTs as a function of 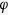 are compared in Fig. 5. We observe that *t*_typ_ is 2–3 orders of magnitude smaller than the corresponding MFPT. Moreover, the amplitude of the FPT distribution at the positions of *t*_typ_ and of the MFPT differs by around one order of magnitude. The MFPT hence is an imprecise measure for the FPT kinetics of Brownian motion in both homogeneous and heterogeneous media. Typical trajectories are hence direct in the sense that they do not reach the external boundary. In fact, provided with the results for the three time scales of the current FPT problem obtained in the previous sections we are now in the position to make a more precise statement: *Typical FPTs are strictly shorter than the average time needed to arrive at the target via reflection from the interface.* Note that this statement does not contradict the case of starting at the reflecting surface, as the first reflection would demand a return to the initial position.

Conversely, it is obvious that while the long time FPT behaviour *per se* is completely independent of the initial particle position, the contribution of this regime to the MFPT depends on the relative time span of the regime in comparison to the short and intermediate time regimes. In other words, the long time regime always has a strictly additive contribution, whose relative magnitude does depend on 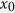 through the lower limit of integration. To see this we can approximately split the MFPT into a long time contribution 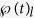 and the remainder 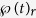, where the respective parts hold for 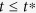 and 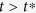, where 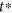 is the lower bound of validity of the long-time asymptotic result. Then from Eqs (7) we find that


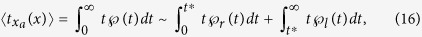


where the second term depends on 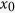 solely through 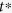. Using Eq. (7) and performing the integral we find that the long time contribution to the MFPT—the second term in Eq. (16)—is always equal to


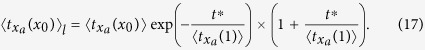


It is straightforward to check that the long time exponential tail dominates the MFPT. This finding is *a priori* puzzling as it appears to be incompatible with the additivity of the MFPT in Eq. (9). This can be resolved from cognisance of the fact that the second integrand is always the exact MFPT scaled by a unit exponential 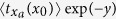, and the integration is over *y* from 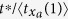 to ∞. It should be noted that for most physically realistic situations we have 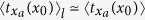. In addition, in the limit 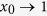 we obtain the lower bound 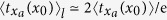. We are hence in the position to make the quite powerful statement, refining the results in ref. 32: *Despite being dominated by the rare long time, indirect trajectories the MFPT is in fact completely specified by the statistics of direct trajectories. Namely, the fraction and duration of direct trajectories rescales the otherwise invariant contribution of indirect ones. In other words, the value of the integral is essentially constant but its overall statistical weight is set by the direct trajectories. This also explains the result (9).* Moreover, the non-monotonic behaviour with respect to 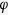 for 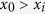 and the corresponding existence of a minimum can be understood intuitively in terms of the balance between the rate to arrive from 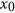 to the interface and the rate to arrive from 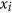 to the target, compare also ref. 32.

We finally quantify the width Δ of the FPT distribution 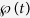. Since we know that very short and very long trajectories are exponentially unlikely, we may use these cutoff times obtain


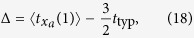


such that the width depends on the initial position solely through 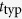. Moreover, we find that


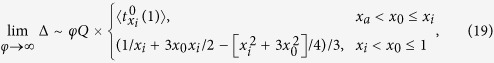


demonstrating that the width increases as 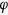 grows and effects a progressive retardation of the dynamics in the outer region.

## Discussion

We analysed a simple prototype model for Brownian motion in heterogeneous environments in terms of a spherically symmetric geometry with two concentric regions of different diffusivity. We obtained rigorous, asymptotically exact results for the FPT distribution and identified a short time Lévy-Smirnov as well as a long time exponential behaviour. Moreover, we demonstrated the existence of a hitherto overlooked intermediate time scale. All three time scales were interpreted in terms of direct and indirect trajectories. The distinction between the latter also effects the major discrepancy between the mean and the most likely FPTs. Cognisance of this difference is important in many systems.

We first focus on the implications of our findings for Brownian motion in spherically symmetric homogeneous media. Our results suggest that the prevailing paradigm of first passage kinetics for Brownian motion^1^,^14^,^15^ becomes even richer. First, as highlighted already in previous works^17^,^18^,^19^ the MFPT is often a rough measure of the first passage kinetics: the FPT distribution is typically positively skewed and therefore asymmetric. According to the results in refs 17, 18, 19 any two arbitrary trajectories are often more likely to be very different than similar. From our results reported here this result is substantiated in the sense that according to our interpretation an arbitrary trajectory will typically be direct and will not interact with the reflecting boundary. For homogeneous Brownian motion this holds strictly for all starting positions satisfying 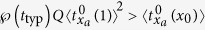. This statement is particularly important for single molecule observations, where a finite number of trajectories will more likely reveal the typical and not the mean behaviour. We have shown that there is a large discrepancy between the typical FPT and the MFPT. Moreover, an upper bound for the FPT—if starting from an arbitrary position—is set by the MFPT from the confining surface. In the latter case, the distinction between direct and indirect trajectories obviously ceases to exist, giving rise to dominantly exponential statistics. Conversely, the MFPT, while indeed dominated by the rare long time behaviour, is remarkably fully specified by the typical direct trajectories. Hence, the most likely but less significant trajectories turn out to determine the unlikely but dominant trajectories. This adds another surprising feature to the first passage behaviour of Brownian motion, starting with the Lévy arcsine laws for one dimensional free Brownian motion^41^,^42^.

We demonstrated that a sufficient heterogeneity in the diffusion coefficient gives rise to an additional, intermediate time scale, on which trajectories contain a short excursion towards the external surface. This excursion, however, is much shorter than the typical time needed to diffuse across the entire domain. In this *heterogeneity-controlled* kinetic domain there thus exist three distinct classes of trajectories: the direct ones, the indirect ones and those which initially make a short indirect excursion and then go directly to the target, as shown in Fig. 1. This latter class of trajectories in fact represents the typical trajectories for starting positions in the outer region of our model system.

The results for our idealised two component model studied in the present work have important consequences for an arbitrary spherically symmetric modulation of the diffusivity. Namely, in such a case there might exist several distinct time scales in the heterogeneity controlled kinetic regime. In fact, heterogeneity controlled kinetics will generically be observed in the presence of sharp changes in the local diffusivity 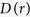. Such sharp modulations are indeed present in cellular signalling processes when a particle starts in the cytoplasm and searches for its target in the nucleus, where the diffusivity is different. In particular, signalling particles synthesised inside the cell as part of a particular signalling cascade will inherently start away from the cellular membrane, and a separation of scales in the FPT is therefore expected to exist. Moreover, as a large discrepancy is expected to exist in the likelihood for observing typical FPTs with respect to the MFPT, the MFPT is a fairly poor measure for the kinetics at low copy numbers of signalling molecules and for a finite number of possible realisations. Conversely, smooth modulations of 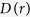 are not expected to change the qualitative two time scale picture of FPT kinetics but will of course alter the coefficients in the short and long time asymptotic regimes. We can extend the discussion also to systems with off-centre targets under the condition that the searching particle does not start too close to the external boundary. In this case the long and intermediate time scale asymptotics would remain unchanged—as long as there exist a separation of scales, of course—but the long time asymptotics would be altered. Thus, our rigorous results for our idealised model system are relevant for FPT kinetics in generic heterogeneous media.

We also briefly comment on alternative forms of the diffusion equation (2), the so called Ito and Stratonovich forms^35^. Here we focus on physical stochastic dynamics satisfying the fluctuation-dissipation theorem. In this view, the Ito and Stratonovich equations are nothing but the corresponding Fokker-Planck equations with diffusion coefficient 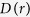 in an external potential 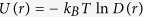 and 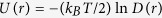, respectively. The MFPT problem in such scenarios including a spurious drift was studied recently in^43^. The FPT kinetics in the presence of such effective external fields will therefore be fundamentally different. We finally note that it will be of further interest to include intermittent active motion in the analysis^44^.

### Explicit expressions for the coefficients 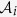 and 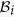


The coefficients in Eq. (11a) read


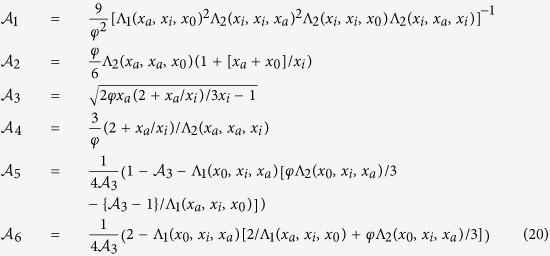


where we have introduced the auxiliary functions


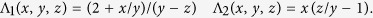


Conversely, the coefficients in Eq. (11a) are


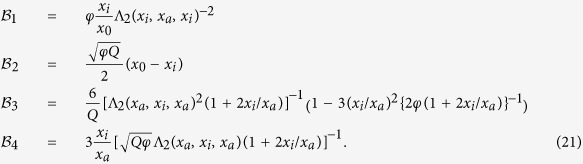


## Additional Information

**How to cite this article**: Godec, A. and Metzler, R. First passage time distribution in heterogeneity controlled kinetics: going beyond the mean first passage time. *Sci. Rep.* **6**, 20349; doi: 10.1038/srep20349 (2016).
